# Single-drug therapy or selective decontamination of the digestive tract as antifungal prophylaxis in critically ill patients: a systematic review

**DOI:** 10.1186/cc6191

**Published:** 2007-12-07

**Authors:** JW Olivier van Till, Oddeke van Ruler, Bas Lamme, Roy JP Weber, Johannes B Reitsma, Marja A Boermeester

**Affiliations:** 1 Department of Surgery, Academic Medical Center, P.O. Box 22660, 1100 DD Amsterdam, The Netherlands; 2 Department of Clinical Epidemiology, Biostatistics and Bioinformatics, Academic Medical Center, Room J1b-208, Meibergdreef 9, 1105 AZ, Amsterdam, the Netherlands

## Abstract

**Introduction:**

The objective of this study was to determine and compare the effectiveness of different prophylactic antifungal therapies in critically ill patients on the incidence of yeast colonisation, infection, candidemia, and hospital mortality.

**Methods:**

A systematic review was conducted of prospective trials including adult non-neutropenic patients, comparing single-drug antifungal prophylaxis (SAP) or selective decontamination of the digestive tract (SDD) with controls and with each other.

**Results:**

Thirty-three studies were included (11 SAP and 22 SDD; 5,529 patients). Compared with control groups, both SAP and SDD reduced the incidence of yeast colonisation (SAP: odds ratio [OR] 0.38, 95% confidence interval [CI] 0.20 to 0.70; SDD: OR 0.12, 95% CI 0.05 to 0.29) and infection (SAP: OR 0.54, 95% CI 0.39 to 0.75; SDD: OR 0.29, 95% CI 0.18 to 0.45). Treatment effects were significantly larger in SDD trials than in SAP trials. The incidence of candidemia was reduced by SAP (OR 0.32, 95% CI 0.12 to 0.82) but not by SDD (OR 0.59, 95% CI 0.25 to 1.40). In-hospital mortality was reduced predominantly by SDD (OR 0.73, 95% CI 0.59 to 0.93, numbers needed to treat 15; SAP: OR 0.80, 95% CI 0.64 to 1.00). Effectiveness of prophylaxis reduced with an increased proportion of included surgical patients.

**Conclusion:**

Antifungal prophylaxis (SAP or SDD) is effective in reducing yeast colonisation and infections across a range of critically ill patients. Indirect comparisons suggest that SDD is more effective in reducing yeast-related outcomes, except for candidemia.

## Introduction

Yeast colonisation is quite common in intensive care unit (ICU) populations. Up to 73% of patients have been reported to be colonised by yeast, predominantly by *Candida albicans *[1]. *Candida *species are among the most commonly isolated microorganisms from the abdomen and urine in surgical patients with infections [2].

The development of fungal/yeast infections is a rapidly increasing health problem, especially in hospitalized patients and in patients with impaired host defences. In 1995, yeast was reported to be the fourth most common ICU-acquired infection in Europe, where it represented approximately 17% of all isolates [3]. This percentage may be even higher now, although more recent data are lacking. Particularly in patients with peritonitis, *Candida *frequently can be cultured from the abdomen, with prevalences as high as 30% to 40% [4-7]. Systemic yeast infections are associated with high mortality, often more than 50% [8], with *C. albicans *as the predominant species responsible [9]. Systemic fungal/yeast infections have become more common over the past two decades. *Candida *is the fourth leading cause of all nosocomial bloodstream infections in the US, accounting for up to 11% of all infections [10]. As early as the 1980s, an increase in surgical yeast infections from 2.5/1,000 discharges to 5.6/1,000 discharges was observed [11]. The incidence of candidemia increased to 9.8/1,000 ICU admissions among postoperative ICU patients in 1999 [12]. In another study, the incidence increased from 1.25/10,000 in 1999 to 3.06/10,000 patient-days per year in 2003 [13]. Invasive yeast infections are associated with high morbidity and mortality, and the cost of bloodstream *Candida *infection alone is already approaching $1 billion per year in the US [14].

Proper management of yeast infections is challenging because the diagnosis is often elusive. At present, laboratory tests can be inconclusive (blood cultures have a sensitivity of only 70% [15]) and it is difficult to distinguish between colonisers and pathogens. Yeast is part of the physiological microbiological flora, thus positive cultures may merely reflect colonisation or environmental contamination instead of actual infection. On the other hand, the gold standard for the diagnosis of candidemia, blood culture, is not perfect. False-negative blood cultures, especially, are a problem because sensitivity is approximately 70% [15].

Given the high and increasing incidence of *Candida *infection, its major clinical impact, and the lack of tests for an early and accurate diagnosis, a prophylactic approach for high-risk patients might be beneficial. Previous reviews on this topic have analysed specific yeast prophylaxis regimens with either a single-drug antifungal prophylaxis (SAP) or a multi-drug regimen of selective decontamination of the digestive tract (SDD). There are no direct randomised comparisons between SAP and SDD treatments. Our aim is to review and compare the effectiveness of both therapeutic strategies on yeast colonisation, invasive yeast infection, candidemia, and in-hospital mortality.

## Materials and methods

### Search strategy

To identify eligible studies, a computer-assisted search was performed in the following medical databases: Medline (January 1966 to January 2006), Cochrane Database of Systematic Reviews, Cochrane Clinical Trials Register, Database of Abstracts on Reviews and Effectiveness, and EMBASE (January 1950 to January 2006). Search terms included 'Candida', 'yeast', 'fungal', 'antimycotic', 'antifungal', 'prophylaxis', 'pre-emptive', 'SDD', and 'SGD' (selective gut decontamination). Clinical studies published in English, German, or French were included. A manual cross-reference search of the eligible papers was performed to identify additional relevant articles. No unpublished data or data from abstracts were included in the review.

### Inclusion and exclusion criteria definitions

Clinical studies were eligible for inclusion if they assessed adult non-neutropenic patients without concurrent immune suppression (due to chemotherapy, solid organ or bone marrow transplantation, neutropenia, or HIV/AIDS) undergoing preventive (pre-emptive or prophylactic) antimycotic therapy with any antifungal agent. Prophylaxis in this review is defined as antifungal therapy without a proven fungal infection. Pre-emptive therapy is defined as antifungal therapy given for a non-proven, but suspected, fungal infection.

Studies were excluded if they were retrospective or if they did not compare the treated patient group with a control group that either received no antifungal therapy or received placebo. Studies examining the effects of antifungal prophylaxis without measuring or reporting the incidence of *Candida *or yeast infection or colonisation were also excluded.

We aimed to retrieve the following outcomes from all studies: (a) yeast colonisation defined as positive yeast culture obtained from sputum, stool, urine, and/or wound without clinical signs of infection/inflammation, (b) invasive yeast infection defined as positive yeast culture obtained from presumed sterile sites (peritoneal cavity, deep tissue, invasive burn wound, or bronchoalveolar lavage fluid) with clinical signs of infection/inflammation, (c) candidemia defined as positive yeast culture from two or more blood cultures, (d) all-cause in-hospital mortality, and (e) mortality directly attributable to yeast infection. The definitions of colonisation and infection varied between individual studies, but results were extracted using the above-mentioned definitions.

The methodological quality of the individual studies was scored using the Jadad scale, rated by one author (JvT). This is a well-known instrument assigning a numerical score between 0 and 5 to each study, reflecting its quality (0 indicating poor quality and 5 high quality) [16]. The research was carried out in compliance with the World Medical Association Declaration of Helsinki [17].

### Statistical analysis

Patient characteristics of included patients are presented as medians with 25% to 75% interquartile range (IQR). The effectiveness of either therapy (SAP or SDD) compared to their control group was expressed using odds ratios (ORs) with 95% confidence intervals (CIs). An OR of less than 1 signifies a reduced risk of developing an adverse outcome in a prophylaxis group compared to controls. Random effects models were used to calculate pooled ORs and 95% CIs across studies. To improve interpretability of results, we also calculated the number needed to treat (NNT). NNT indicates the number of patients who have to be treated with antifungal prophylactic treatment in order to avoid one adverse outcome. NNT was calculated by taking the reciprocal of the risk difference, which is the absolute arithmetic difference in rates of outcomes between treated and control participants. Studies were heterogeneous when more variation between the study results was observed than would be expected to occur by chance alone. Heterogeneity in results across studies was assessed by the Q test with κ – 1 degrees of freedom (DFs) and by calculating I^2^. I^2 ^is a measure of inconsistency describing the percentage of total variation across studies that is due to heterogeneity rather than chance.

### Analysis strategy

Firstly, pooled ORs for SAP and SDD studies were calculated separately. A formal test of interaction (meta-regression model to test the null hypothesis that the difference in random effects pooled ORs of SAP versus SDD studies is zero) was performed to determine whether there was evidence that the pooled OR was different between SAP and SDD studies. If there was no indication for a treatment difference (*p *value of interaction test above 0.1), a summary OR was calculated combining SAP and SDD studies. In an additional analysis, it was examined whether the proportion of surgical patients could have influenced the observed differences in effectiveness between SAP and SDD studies, because it has been suggested that surgical patients are specifically at risk of developing a yeast infection [18] and would benefit most from antifungal therapy [19]. The effect of the proportion of surgical patients as a confounder on outcomes was assessed by comparing crude relative OR (crude OR SAP divided by crude OR SDD) and relative OR adjusted for the proportion of surgical patients (adjusted OR SAP divided by adjusted OR SDD). The proportion of surgical patients included was regarded as a confounder when a difference of 10% or more between crude and adjusted relative ORs was found. Within the group of SAP studies, it was also examined whether systemic (absorbable) drugs were more or less effective than non-absorbable enteral antifungal drugs, comparing the crude and adjusted ORs using logistic regression.

Because of the risk of publication bias, a 'failsafe N' was calculated for the meta-analyses with significantly positive outcomes. This number denotes the number of studies with null results that would need to be added to the meta-analysis in order for an effect to no longer be reliable, the so-called 'file drawer studies' [20,21]. The magnitude of this sample is a measure for the validity of the conclusions of the analyses in this review.

Data analysis was performed using Review Manager 4.2.8 software (The Cochrane Collaboration, Oxford, Oxfordshire, UK), SAS (Statistical Analysis System) software version 9.1 (SAS Institute Inc., Cary, NC, USA), and Statistical Package for the Social Sciences version 11.5 (SPSS Inc., Chicago, IL, USA). All *p *values were two-sided, with *p *values less than 0.05 indicating statistical significance.

## Results

### Studies

**Figure 1:**
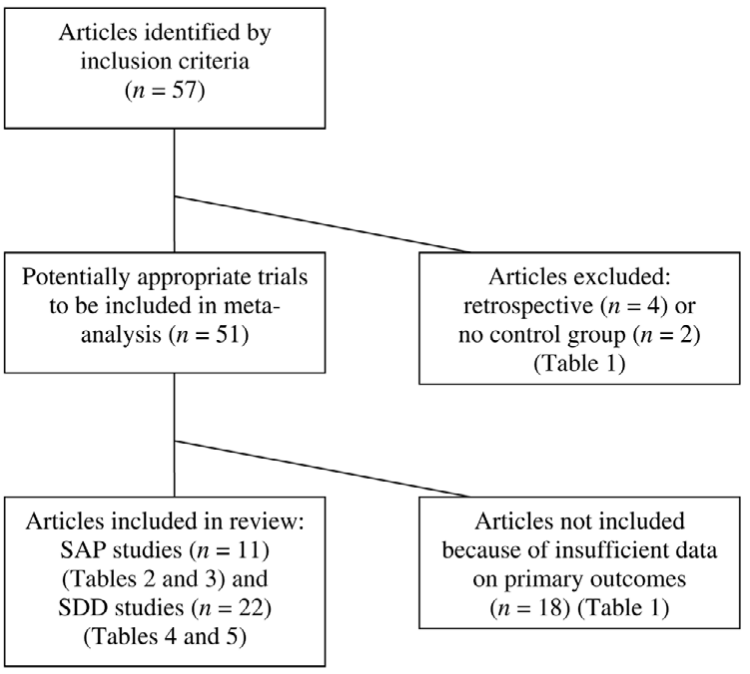
Flowchart showing study inclusion and exclusion. SAP, single-drug antifungal prophylaxis; SDD, selective decontamination of the digestive tract.

In all, 57 clinical studies examined either the SAP or SDD regimen in adult patients (Figure 1). No studies directly comparing SAP versus SDD were found. Twenty-four of these studies were excluded. Table 1 presents the reasons for exclusion: the studies did not report on *Candida*/yeast/fungus infection/colonisation or fungemia/candidemia (*n *= 14), they reported percentages of positive *Candida *cultures among cultures instead of among patients (*n *= 4), they had a retrospective design (*n *= 4), or they had no control group (*n *= 2). Therefore, a total of 33 prospective studies were included in this review: 11 studies examining the effects of SAP and 22 studies on SDD. Study and patient characteristics are presented in Tables 2 and 3 for SAP and in Tables 4 and 5 for SDD. Table 6 presents a summary of outcome parameters reported in the 33 included studies.

**Table 1:** Excluded studies examining antifungal prophylaxis in adult non-neutropenic patients (*n *= 24)

Reference	Year	Endpoint	Exclusion	Design
Stoutenbeek *et al*. [41]	1984	SDD in ICU patients	Only yeast as percentage of cultures	Cohort
Slotman *et al*. [42]	1988	Ketoconazole prophylaxis in ALI/ARDS	No yeast scored	RCT
Flaherty *et al*. [43]	1990	SDD in ICU patients	No yeast scored	RCT
Godard *et al*. [44]	1990	SDD in ICU patients	No yeast scored (+ no control group)	RCT
Rodriguez-Roldán *et al*. [45]	1990	SDD in ICU patients	No yeast scored	RCT
Tetteroo *et al*. [46]	1990	SDD in oesophageal resection	No yeast scored	RCT
Gastinne *et al*. [47]	1992	SDD in ICU patients	No yeast scored	RCT
Jacobs *et al*. [48]	1992	SDD in ICU patients	No yeast scored	RCT
Rocha *et al*. [49]	1992	SDD in ICU patients	No yeast scored	RCT
Korinek *et al*. [50]	1993	SDD in neurosurgical ICU patients	Only yeast as percentage of cultures	RCT
Yu and Tomasa [51]	1993	Ketokonazole prophylaxis in ARDS	No yeast scored	RCT
Misset *et al*. [52]	1994	SDD in ICU patients	No control group	RCT
Sorkine *et al*. [53]	1996	Amphotericin B treatment in *Candida *sepsis	No control group	RCT
Lingnau *et al*. [54]	1997	SDD in multiple trauma patients	No yeast scored	RCT
Palomar *et al*. [55]	1997	SDD in ICU patients	No yeast scored	RCT
Safran and Dawson [56]	1997	Fluconazole prophylaxis in SICU patients	Retrospective (+ no control group)	Cohort
Schardey *et al*. [57]	1997	SDD in total gastrectomy	Only yeast as percentage of cultures	RCT
Sánchez García *et al*. [58]	1998	SDD in ICU patients	Only yeast as percentage of cultures	RCT
ARDS network [59]	2000	Ketoconazole prophylaxis in ALI/ARDS	No yeast scored	RCT
Pneumatikos *et al*. [60]	2002	Subglottal decontamination	No yeast scored	RCT
De Waele *et al*. [61]	2003	Fluconazole prophylaxis in pancreatitis	Retrospective	Cohort
Swoboda *et al*. [62]	2003	Fluconazole prophylaxis in SICU patients	Retrospective	Cohort
Magill *et al*. [63]	2004	Fluconazole prophylaxis in SICU patients	No yeast scored	RCT
Shan *et al*. [64]	2006	Early presumptive therapy in GI surgery	Retrospective	Cohort

Reasons for exclusion: no yeast outcomes reported (*n *= 14), only yeast as percentage of cultures (*n *= 4), no control group (*n *= 2), retrospective design (*n *= 4). ALI, acute lung injury; ARDS, acute respiratory distress syndrome; GI, gastrointestinal; ICU, intensive care unit; RCT, randomised controlled trial; SDD, selective decontamination of the digestive tract; SICU, surgical intensive care unit.

**Table 2:** General characteristics of individual studies on prophylactic antifungal therapy using a single-drug antifungal prophylaxis regimen

Reference	Year	Country	Inclusion period	Study design
Slotman and Burchard [65]	1987	USA	October 1982 to July 1985	RCT
Savino *et al*. [25]	1994	USA	July 1990 to December 1991	RCT
Eggimann *et al*. [4]	1999	Switzerland	Not specified	RCT
Ables *et al*. [66]	2000	USA	October 1994 to December 1996	RCT
Pelz *et al*. [67]	2001	USA	January 1998 to January 1999	RCT
Sandven *et al*. [6]	2002	Norway	March 1994 to June 1995	RCT
Garbino *et al*. [68]	2002	Switzerland	30 months	RCT
He *et al*. [69]	2003	China	January 1998 to December 2002	RCT
Jacobs *et al*. [70]	2003	Saudi Arabia	December 1998 to June 2001	RCT
Piarroux *et al*. [38]	2004	France	August 1998 to July 2000	Intervention study
Normand *et al*. [24]	2005	France	February 2002 to July 2002	RCT

RCT, randomised controlled trial.

**Table 3:** Clinical and design characteristics of individual studies on single-drug antifungal prophylaxis

Reference	Inclusion	Patients	Group	
			Intervention (*n*)	Control (*n*)

Slotman and Burchard [65]	SICU, ≥3 risk factors for candidemia	Surgical	Ketoconazole, 200 mg, 1 dd oral (27)	Placebo (30)
Savino *et al*. [25]	Remaining or expected SICU stay of >48 hours	Surgical/Trauma	A. Clotrimazole, 10 mg, 3 dd oral (80)	No prophylaxis (72)
			B. Ketoconazole, 200 mg, 1 dd oral (65)	
			C. Nystatin, 2 × 10^6 ^U, 4 dd oral (75) (total 220)	
Eggimann *et al*. [4]	Recurrent GI perforation or anastomotic leakage	Surgical	Fluconazole, 400 mg, 1 dd iv (23)	Placebo (20)
Ables *et al*. [66]	Expected SICU stay of >48 hours + risk factor for candidiasis	Surgical/Trauma	Fluconazole, 400 mg, 1 dd oral/iv (60)	Placebo (59)
Pelz *et al*. [67]	SICU stay of ≥3 days	Surgical	Fluconazole, 400 mg, 1 dd oral (130)	Placebo (130)
Sandven *et al*. [6]	Confirmed intra-abdominal perforation	Surgical	Fluconazole, 400 mg, 1× peroperative iv (53)	Placebo (56)
Garbino *et al*. [68]	SICU stay of ≥3 days + mechanical ventilation for >48 hours	Surgical/Medical	Fluconazole, 100 mg, 1 dd iv (103)	Placebo (101)
He *et al*. [69]	Severe pancreatitis	Surgical	Fluconazole, 100 mg, 1 dd iv (22)	No prophylaxis (23)
Jacobs *et al*. [70]	ICU patients + septic shock	Surgical/Medical	Fluconazole, 200 mg, 1 dd iv (32)	Placebo (39)
Piarroux *et al*. [38]^a^	Colonisation index of ≥0.4, SICU stay of ≥5 days	Surgical/Trauma	Fluconazole, 400 mg, 1 dd iv (478)	No prophylaxis (455)
Normand *et al*. [24]	Mechanical ventilation for >48 hours	Surgical/Medical	Nystatin, 10^6 ^U, 3 dd oral (51)	No prophylaxis (47)

^a^Treatment design is pre-emptive. dd, daily dose; GI, gastrointestinal; ICU, intensive care unit; iv, intravenous; RCT, randomised controlled trial; SICU, surgical intensive care unit.

**Table 4:** General characteristics of individual studies on prophylactic antifungal therapy as a part of a selective decontamination of the digestive tract regimen

Reference	Year	Country	Inclusion period	Study design
Unertl *et al*. [71]	1987	Germany	May 1984 to January 1985	RCT
Ledingham *et al*. [72]	1988	UK	July 1985 to November 1986	Cohort
Kerver *et al*. [73]	1988	The Netherlands	January 1985 to May 1986	RCT
Von Hünefeld [22]	1989	Germany	1987 to 1989	RCT
Ulrich *et al*. [74]	1989	The Netherlands	October 1986 to September 1987	RCT
McClelland *et al*. [75]	1990	UK	January 1987 to December 1987	Cohort
Hartenauer *et al*. [76]	1990	Germany	1989 to 1990	Non-random CT
Gaussorgues *et al*. [77]	1991	France	September 1988 to September 1991	RCT
Blair *et al*. [78]	1991	UK	September 1988 to January 1990	RCT
Aerdts *et al*. [79]	1991	The Netherlands	May 1985 to September 1987	RCT
Cerra *et al*. [80]	1992	USA	Not specified	RCT
Hammond *et al*. [81]	1992	South Africa	January 1989 to December 1990	RCT
Saunders *et al*. [82]	1994			
Cockerill *et al*. [83]	1992	USA	1986 to 1989	RCT
Winter *et al*. [84]	1992	UK	22 months	RCT
Ferrer *et al*. [85]	1994	Spain	Not specified	RCT
Langlois-Karaga *et al*. [86]	1995	France	2 years	RCT
Luiten *et al*. [87]	1995	The Netherlands	April 1990 to April 1993	RCT
Wiener *et al*. [88]	1995	USA	8 months	RCT
Quinio *et al*. [89]	1996	France	Not specified	RCT
Verwaest *et al*. [90]	1997	Belgium	19 months	RCT
Abele-Horn *et al*. [91]	1997	Germany	Not specified	RCT
de La Cal *et al*. [92]	2005	Spain	May 1997 to January 2000	RCT

CT, controlled trial; RCT, randomised controlled trial.

**Table 5:** Clinical and design characteristics of individual studies on selective decontamination of the digestive tract

Reference	Inclusion	Patients	Group	
			Interventions (*n*)	Control (*n*)

Unertl *et al*. [71]	Expected mechanical ventilation of >6 days	Surgical/Trauma/Medical	Amphotericin B, 300 mg, 4 dd oral (19)	Placebo (20)
Ledingham *et al*. [72]	All ICU patients	Surgical/Medical	Amphotericin B, 500 mg, 4 dd oral (163)	No prophylaxis (161)
Kerver *et al*. [73]	ICU stay of >5 days + mechanical ventilation	Surgical/Trauma	Amphotericin B, 500 mg, 4 dd oral (49)	No prophylaxis (47)
Von Hünefeld [22]	Mechanical ventilation for >4 days	Surgical/Trauma	Amphotericin B, 500 mg, 4 dd oral (102)	No prophylaxis (102)
Ulrich *et al*. [74]	Expected ICU stay of >5 days	Surgical/Trauma/Medical	Amphotericin B, 500 mg, 4 dd oral (48)	Placebo (52)
McClelland *et al*. [75]	Acute respiratory and renal failure, mechanical ventilation and hemodialysis for >5 days	Surgical/Trauma/Medical	Amphotericin B, 500 mg, 4 dd oral (15)	No prophylaxis (12)
Hartenauer *et al*. [76]	Mechanical ventilation for >3 days, ICU stay of >5 days	Surgical/Trauma	Amphotericin B, 500 mg, 4 dd oral (99)	No prophylaxis (101)
Gaussorgues *et al*. [77]	Mechanical ventilation + inotropic therapy	Surgical/Medical	Amphotericin B, 500 mg, 4 dd oral (59)	Placebo (59)
Hammond *et al*. [81]	Expected mechanical ventilation of >48 hours, expected ICU stay of >5 days	Surgical/Trauma/Medical	Amphotericin B, 500 mg, 4 dd oral (114)	Placebo (125)
Cockerill *et al*. [83]	ICU stay of ≥3 days	Surgical/Trauma/Medical	Nystatin, 10^5 ^U, 4 dd oral (75)	No prophylaxis (75)
Winter *et al*. [84]	ICU stay of >2 days	Surgical/Trauma/Medical	Amphotericin B, 500 mg, 4 dd oral (91)	No prophylaxis (92)
Ferrer *et al*. [85]	Expected mechanical ventilation of >3 days	Surgical/Trauma/Medical	Amphotericin B, 500 mg, 4 dd oral (39)	Placebo (40)
Langlois-Karaga *et al*. [86]	ICU stay of >2 days	Trauma	Amphotericin B, 500 mg, 4 dd oral (47)	Placebo (50)
Luiten *et al*. [87]	Severe pancreatitis	Surgical/Medical	Amphotericin B, 500 mg, 4 dd oral (50)	No prophylaxis (52)
Wiener *et al*. [88]	Expected mechanical ventilation of >48 hours	Surgical/Medical	Nystatin, 10^5 ^U, 4 dd oral (30)	Placebo (31)
Quinio *et al*. [89]	ICU patients + mechanical ventilation	Trauma	Amphotericin B, 500 mg, 4 dd oral (76)	Placebo (72)
Verwaest *et al*. [90]	Expected mechanical ventilation of >48 hours	Surgical/Trauma	Amphotericin B, 500 mg, 4 dd oral (393)	No prophylaxis (185)
Abele-Horn *et al*. [91]	Mechanical ventilation for >48 hours	Surgical/Medical	Amphotericin B, 500 mg, 4 dd oral (58)	No prophylaxis (30)
de La Cal *et al*. [92]	≥20% of body surface burned, inhalation trauma + ICU stay of ≥3 days	Trauma	Amphotericin B, 500 mg, 4 dd oral (53)	Placebo (54)

dd, daily dose; ICU, intensive care unit; RCT, randomised controlled trial.

**Table 6:** Summary of outcomes presented in included studies

Reference	Colonisation	Infection	Candidemia	Mortality	Attributable mortality
Abele-Horn *et al*. [91]	x			x	
Ables *et al*. [66]		x		x	
Aerdts *et al*. [79]	x	x	x	x	
Blair *et al*. [78]		x	x	x	
Cerra *et al*. [80]		x	x	x	
Cockerill *et al*. [83]		x	x	x	
de La Cal *et al*. [92]			x	x	
Eggimann *et al*. [4]	x	x		x	x
Ferrer *et al*. [85]	x	x		x	x
Garbino *et al*. [68]	x	x	x	x	x
Gaussorgues *et al*. [77]			x	x	
Hammond *et al*. [81], Saunders *et al*. [82]	x	x		x	
Hartenauer *et al*. [76]		x	x	x	
He *et al*. [69]		x		x	
Jacobs *et al*. [70]		x	x	x	
Kerver *et al*. [73]	x			x	
Langlois-Karaga *et al*. [86]		x			
Ledingham *et al*. [72]		x		x	
Luiten *et al*. [87]		x		x	
McClelland *et al*. [75]	x	x		x	
Normand *et al*. [24]	x	x	x	x	
Pelz *et al*. [67]		x	x	x	
Piarroux *et al*. [38]		x	x	x	x
Quinio *et al*. [89]			x	x	
Sandven *et al*. [6]		x		x	
Savino *et al*. [25]	x		x	x	
Slotman and Burchard [65]	x	x		x	x
Ulrich *et al*. [74]	x	x	x	x	
Unertl *et al*. [71]	x			x	
Verwaest *et al*. [90]		x		x	
Von Hünefeld [22]	x		x	x	x
Wiener *et al*. [88]	x	x	x	x	
Winter *et al*. [84]		x	x	x	

In the analysis of SAP studies, 10 randomised controlled trials (RCTs) and 1 prospective intervention study with a historical control group were included (Table 2). In the analysis of SDD studies, 19 RCTs, 2 prospective cohort studies, and 1 non-randomised placebo-controlled study were included (Table 4). The median quality score of the RCTs was good: 3.5 (IQR 3 to 5) for the SAP studies and 3 (IQR 2 to 4) for the SDD studies. Of the 29 RCTs, 20 described the method of randomisation and 15 were double-blinded. In 1 study, the randomisation method was inappropriate [22].

A total of 5,529 patients were analysed: 2,947 patients received antifungal prophylaxis (1,199 in SAP studies and 1,748 in SDD studies) and 2,582 controls received no prophylaxis (1,032 in SAP studies and 1,550 in SDD studies). The general characteristics did not differ between treated patients and control patients. The median age was 55 (IQR 48 to 59) years, median proportion of females 38% (IQR 31% to 43%), median APACHE II (Acute Physiology and Chronic Health Evaluation II) score 16 (IQR 13 to 19), and median proportion of surgical patients 49% (IQR 24% to 77%).

Risk factors known to be independently associated with *Candida *infection and candidemia in multivariate analysis (APACHE II, corticosteroid use, colonisation intensity, renal failure/hemodialysis, total parenteral nutrition, and central venous catheter [18,23]) were surveyed. When reported, there were no significant differences between treated patents and controls or between SAP and SDD groups. However, very few studies actually reported these factors, and no firm conclusions can be drawn. The same was encountered when the contribution of concurrent antibiotic or corticosteroid therapy was examined.

Comparison of pooled characteristics between SAP and SDD groups showed no significant differences, except for the proportion of surgical patients, which was a median of 73% (IQR 43% to 100%) in the SAP group versus 43% (IQR 17% to 62%) in the SDD group (*p *= 0.016). The proportion of surgical patients approximated the percentage of patients who underwent a laparotomy at the least. However, several studies presented only the proportion of surgical patients, without specifying the procedure. It was not possible to compare gastrointestinal surgery with other surgery.

### Colonisation

**Figure 2:**
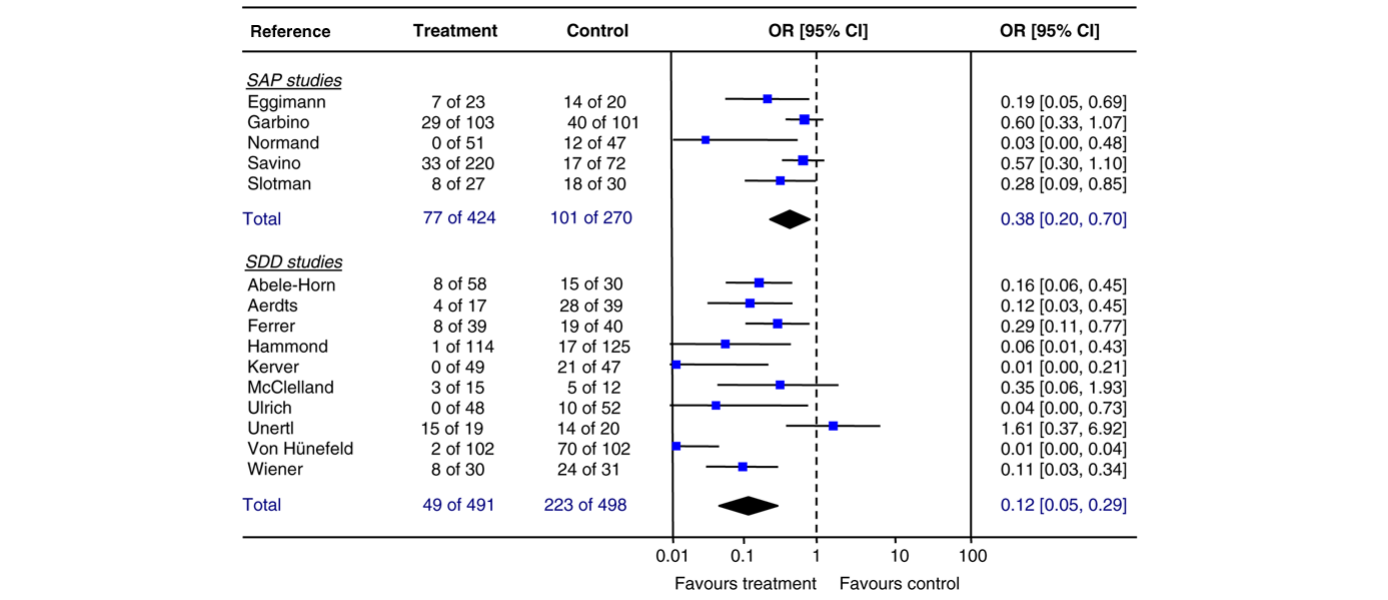
**Yeast colonisation**. Individual and pooled odds ratios (ORs) for yeast colonisation from studies comparing single-drug antifungal prophylaxis (SAP) versus control (upper part) and selective decontamination of the digestive tract (SDD) versus control (lower part) in adult non-neutropenic patients. The model used is a random effects meta-analysis. Test for overall effect: SAP: Z = 3.09 (*p *= 0.002); SDD: Z = 4.58 (*p *< 0.001). Difference in pooled ORs between SAP and SDD studies, test for interaction *p *= 0.020. CI, confidence interval.

Fifteen studies (5 out of 11 SAP studies and 10 out of 22 SDD studies) published data on yeast colonisation (Table 6). Pooled data showed a highly significant reduction of the risk of colonisation for both prophylactic therapies (SAP studies: pooled OR 0.38, 95% CI 0.20 to 0.70, NNT 5; SDD studies: pooled OR 0.12, 95% CI 0.05 to 0.29, NNT 3) (Figure 2). Non-significant heterogeneity was seen for the SAP studies (DF = 4, *p *= 0.10, I^2 ^= 48.7%), whereas heterogeneity was indeed found for the SDD studies (DF = 9, *p *< 0.001, I^2 ^= 73.5%). Both SAP and SDD reduced colonisation: from 37% to 18% in SAP and from 45% to 10% in SDD. The difference between the ORs of SAP and SDD was significant (test for interaction *p *= 0.020), with the effect in SDD studies being 3.6 times higher than in SAP studies (relative OR 3.62, 95% CI 1.12 to 11.77). The failsafe N values for SAP and SDD were 25 and 194, respectively.

### Invasive infection

**Figure 3:**
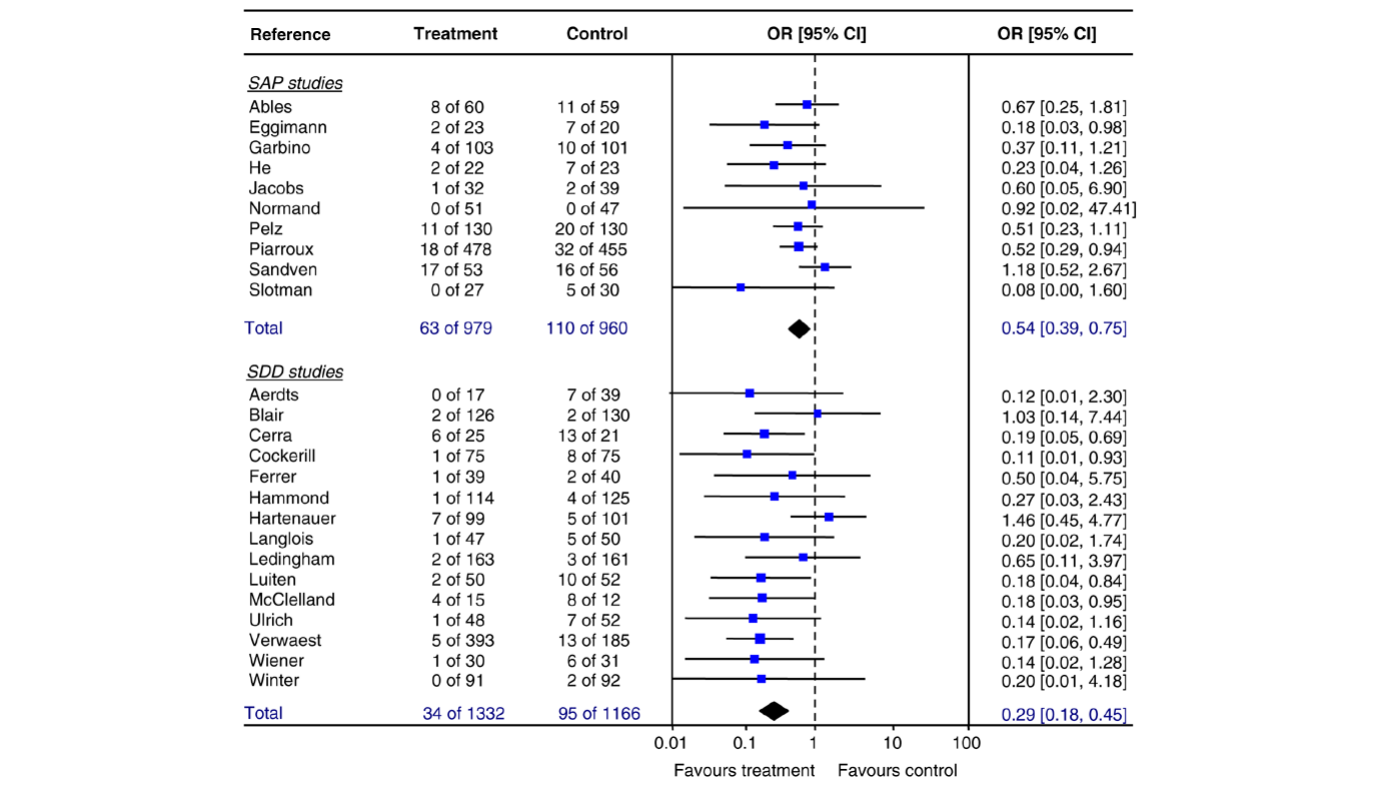
**Invasive yeast infection**. Random effects meta-analysis of the effect of single-drug antifungal prophylaxis (SAP) and selective decontamination of the digestive tract (SDD) on invasive yeast infection (per patient) in adult non-neutropenic patients. Test for overall effect: SAP: Z = 3.44 (*p *< 0.001); SDD: Z = 5.28 (*p *< 0.001). Difference in pooled odds ratios (ORs) between SAP and SDD studies, test for interaction *p *= 0.036. CI, confidence interval.

Data on invasive yeast infection were available from 25 studies (10 SAP studies and 15 SDD studies) (Table 6). A significant reduction of the risk of invasive infection was found (Figure 3) for SAP studies with a pooled OR of 0.54 (95% CI 0.39 to 0.75, NNT 20) and for SDD studies with a pooled OR of 0.29 (95% CI 0.18 to 0.45, NNT 18). Heterogeneity of included studies was not significant for either set of studies (SAP: DF = 8, *p *= 0.40, I^2 ^= 3.8%; SDD: DF = 14, *p *= 0.45, I^2 ^= 0%).

The effect on yeast infection was significantly more pronounced in SDD studies than in SAP studies (test for interaction *p *= 0.036; relative OR 2.0, 95% CI 1.1 to 3.7). SDD reduced the incidence of invasive infection from 8% in control patients to 3% in prophylaxis patients. The failsafe N values for SAP and SDD were 26 and 101, respectively.

It was not possible to determine the ability of the preventative therapies to prevent pure invasive yeast infection unencumbered by concomitant bacterial infection (polymicrobial infections), as most studies did not provide data on concurrent microbial cultures. Too few studies reported data on colonisation and infection of specific infected body sites to draw a conclusion on which body sites principally benefited from antifungal prophylaxis.

### Candidemia

**Figure 4:**
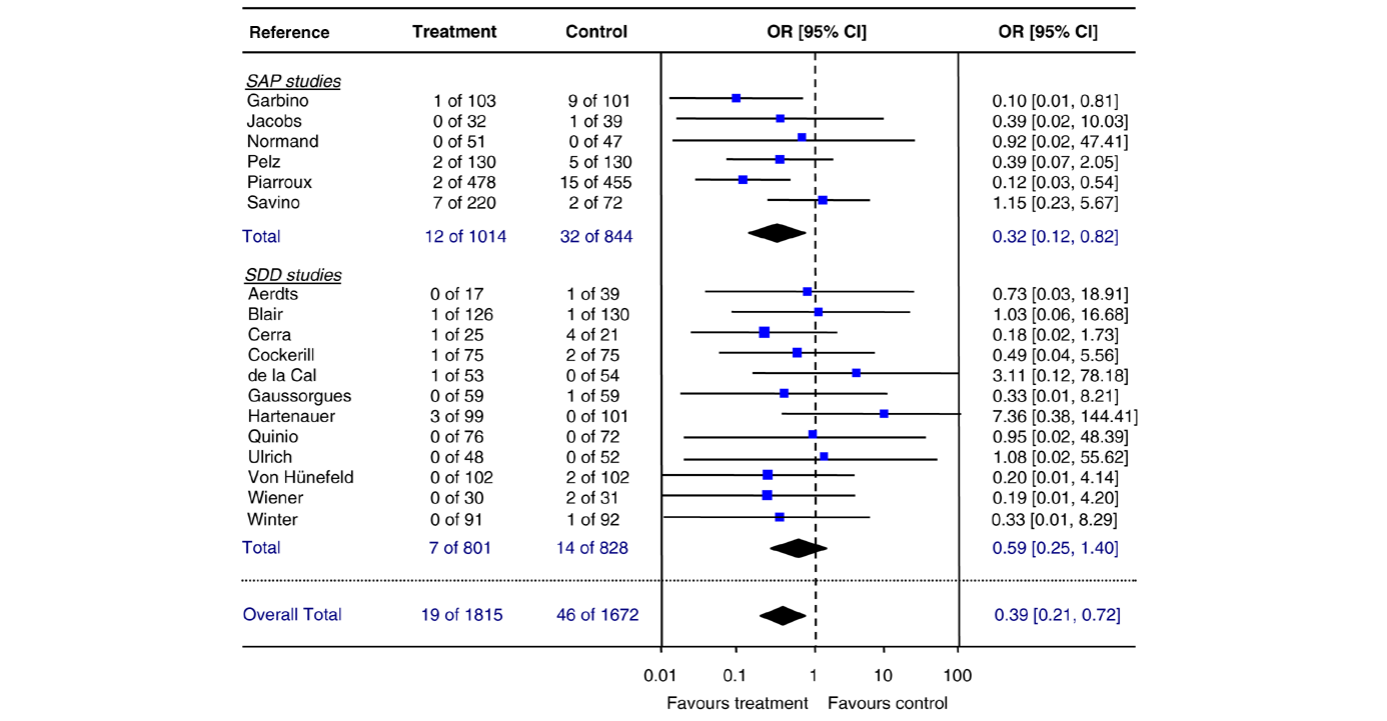
**Candidemia**. Random effects meta-analysis of the effect of single-drug antifungal prophylaxis (SAP) and selective decontamination of the digestive tract (SDD) on candidemia (per patient) in adult non-neutropenic patients. Test for overall effect: SAP: Z = 2.47 (*p *= 0.01); SDD: Z = 1.26 (*p *= 0.21); both groups combined: Z = 3.04 (*p *= 0.002). Difference in pooled odds ratios (ORs) between SAP and SDD studies, test for interaction *p *= 0.34. CI, confidence interval.

Data on candidemia were published in 18 studies (6 SAP studies and 12 SDD studies) (Table 6). The analysis of SAP studies showed a significant reduction of the risk of candidemia by prophylactic therapy (pooled OR 0.32, 95% CI 0.12 to 0.82, NNT 38) (Figure 4), reducing the incidence of candidemia from 3.8% in controls to 1.2% in treated patients. The pooled OR for SDD studies was 0.59, with a wide CI that included 1 (95% CI 0.25 to 1.40) (Figure 4). Heterogeneity of included studies was not significant for SAP studies (DF = 4, *p *= 0.25, I^2 ^= 26.4%) or for SDD studies (DF = 9, *p *= 0.71, I^2 ^= 0%). A formal test of interaction for a difference in treatment effect (pooled ORs) between SAP and SDD studies was not statistically significant (*p *= 0.34), and the overall pooled OR across all 18 studies was 0.39 (95% CI 0.21 to 0.72, NNT 59). The failsafe N for the SAP group was 25.

### Mortality

**Figure 5:**
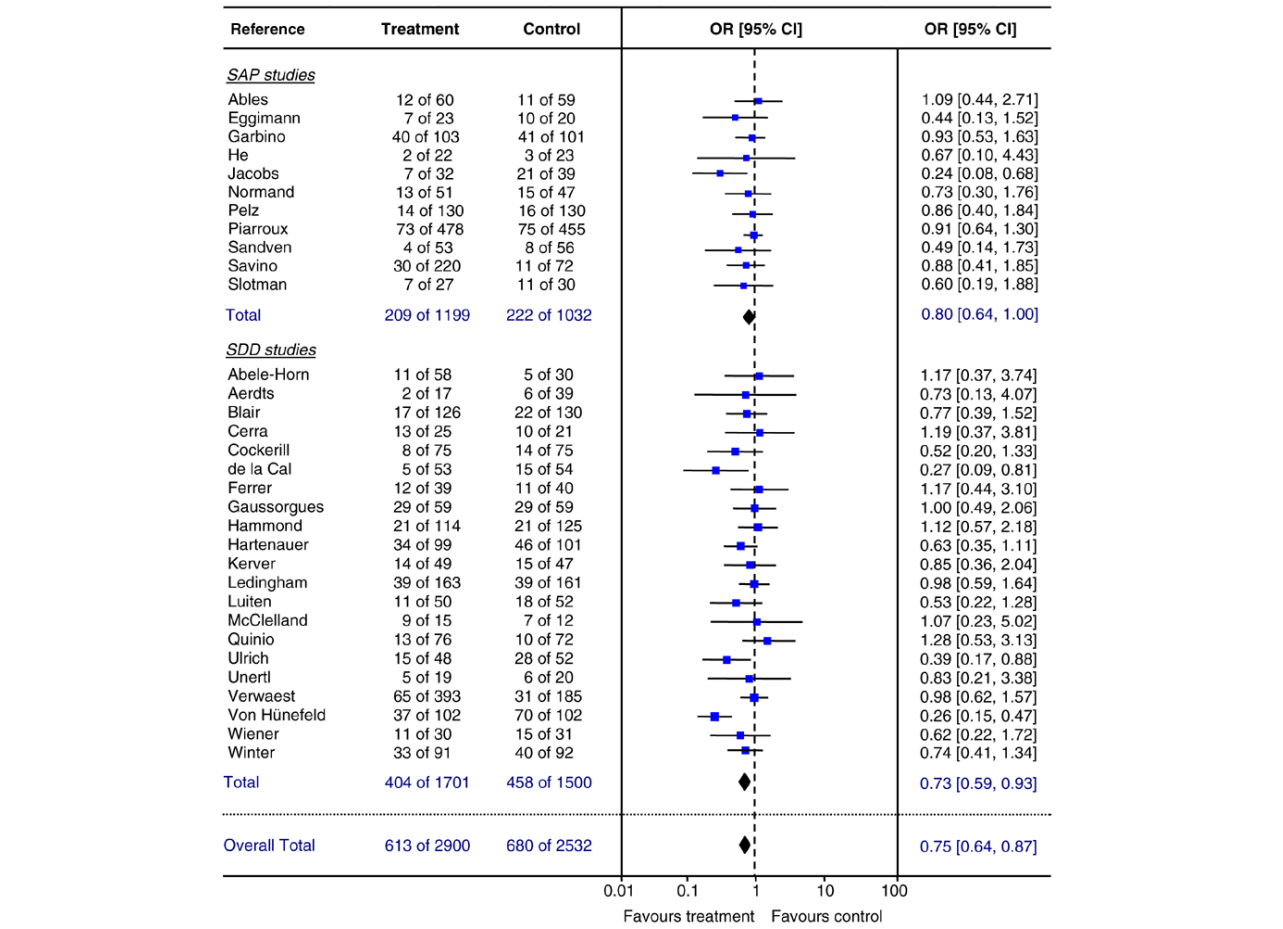
**In-hospital mortality**. Random effects meta-analysis of the effect of single-drug antifungal prophylaxis (SAP) and selective decontamination of the digestive tract (SDD) on all-cause in-hospital mortality (per patient) in adult non-neutropenic patients. Test for overall effect: SAP: Z = 1.97 (*p *= 0.05); SDD: Z = 2.92 (*p *= 0.004); both groups combined: Z = 3.72 (*p *< 0.001). Difference in pooled odds ratios (ORs) between SAP and SDD studies, test for interaction *p *= 0.58. CI, confidence interval.

Data on all-cause hospital mortality were published in 32 studies (all 11 SAP studies and 21 SDD studies) (Table 6). For SAP studies, a pooled OR of 0.80 (95% CI 0.64 to 1.00) was found, whereas the pooled OR for SDD studies was 0.73 (95% CI 0.59 to 0.93, NNT 15) (Figure 5). No heterogeneity of included studies was found either for SAP studies (DF = 10, *p *= 0.61, I^2 ^= 0%) or for SDD studies (DF = 20, *p *= 0.10, I^2 ^= 29.1%).

The pooled ORs of SAP and SDD studies were not significantly different (test for interaction *p *= 0.58). The pooled OR across all 32 studies was 0.75 (95% CI 0.64 to 0.87, NNT 17). Prophylactic antifungal drug administration, being either SAP or SDD, reduced mortality from 27% in controls to 21% in treated patients. The failsafe N for the SDD group was 41. Mortality directly attributable to yeast infection was studied in 6 studies (4 SAP and 2 SDD). Attributable mortality was significantly reduced by prophylaxis: 0.5% in the prophylaxis group versus 2.9% in the control group (pooled OR: 0.23, 95% CI 0.09 to 0.60, NNT 41).

The outcomes of studies with small sample size are more hampered by play of chance than in large sized studies. When studies of fewer than 50 patients or fewer than 100 patients were excluded to exclude noise due to publication bias of small-sample-size positive trials, ORs did not tend to change significantly, except for the ORs of candidemia in the SDD group, which were higher (0.69 [95% CI 0.26 to 1.87] and 0.81 [95% CI 0.26 to 2.46] for exclusion of studies fewer than 50 and fewer than 100 patients, respectively). This underlines the already moderate non-significant effect of SDD on candidemia.

### Additional analyses

#### Surgical patients

Adjustment for the proportion of surgical patients in single antifungal drug and SDD studies changed the difference in pooled ORs between single-drug and SDD studies. Specifically, the proportion of surgical patients was a confounder to the outcomes of colonisation and candidemia. The proportion of surgical patients, on average, was higher in SAP studies compared with SDD studies, as was shown by a reduction of OR after adjustment in the SAP group (colonisation: SAP, crude OR 0.41 [95% CI 0.24 to 0.68] to adjusted OR 0.30 [95% CI 0.11 to 0.86]; SDD, crude OR 0.12 [95% CI 0.05 to 0.29] to adjusted OR 0.12 [95% CI 0.04 to 0.36]; candidemia: SAP, crude OR 0.32 [95% CI 0.12 to 0.82] to adjusted OR 0.16 [95% CI 0.06 to 0.44]; SDD, crude OR 0.59 [95% CI 0.25 to 1.40] to adjusted OR 0.78 [95% CI 0.27 to 2.22]).

After adjustment of the crude relative OR (OR SAP/OR SDD) for the proportion of surgical patients, the adjusted relative OR was significantly reduced for colonisation (crude relative OR 3.62, adjusted relative OR 2.53) as well as for candidemia (crude relative OR 0.51, adjusted relative OR 0.21). Thus, even after adjustment for confounding, the effect found in SDD studies was still significantly different from that found in SAP studies. For yeast infection and mortality, crude and adjusted relative ORs were comparable.

#### Absorbable (systemic) versus non-absorbable (enteral) antifungal drugs

All SDD studies applied non-absorbable drugs, so no comparison could be made within these studies. In three SAP studies, patients received non-absorbable drugs: two studies applied oral nystatin [24,25] and one study applied oral clotrimazole [25]. Only one of these studies reported data on invasive infection, thus no analysis could be performed concerning this outcome.

ORs for systemic and enteral drugs were comparable for colonisation but differed for candidemia. Candidemia was significantly reduced by systemic SAP (OR 0.22, 95% CI 0.09 to 0.52) but not by non-absorbable SAP (OR 1.36, 95% CI 0.37 to 5.03). This difference between ORs of candidemia between groups was significant (test for interaction *p *= 0.022). For mortality, the OR in studies with systemic drugs was 0.75 (95% CI 0.57 to 0.98) and in studies using non-absorbable drugs was 0.97 (95% CI 0.58 to 1.61) (test for interaction *p *= 0.37).

## Discussion

Both methods of antifungal prophylaxis (SAP and SDD) reduced the odds of developing *Candida *colonisation, invasive infection, candidemia, and mortality to various degrees in critically ill patients. The present comparative meta-analysis of SAP and SDD antifungal prophylactic regimens allowed us to analyse the differences between both antifungal strategies on outcomes. This is important since no head-to-head comparison studies have been performed.

SDD was more effective in reducing yeast colonisation and infection than single-drug prophylaxis. The clinical importance of these effects on outcomes is illustrated by the fact that *Candida *colonisation or infection with an identical strain frequently precedes advanced (bloodstream) infection in non-neutropenic patients [26,27]. Furthermore, the intensity of *Candida *colonisation is an independent factor for the development of *Candida *infection [23]. However, in a large prospective cohort study, prior colonisation was not associated with bloodstream infections [18]. The exact role of colonisation in yeast-related disease should be elaborated further to fully appreciate the effects of prophylactic therapy.

The risk of developing yeast infection was reduced 3.2-fold (from 8.3% to 2.6%) by SDD, which was significantly more effective than SAP. Since most SAP regimens comprise absorbable (mostly intravenous) drugs and SDD contains enteral non-absorbable drugs, these results corroborate the hypothesis that the gastrointestinal tract is the primary source of yeast causing infection. Alterations in the host defence of critically ill patients (prior surgery, parenteral nutrition, hemodialysis, and mechanical ventilation) imply immunosuppression and/or a breach of the mucosal barrier [18,23,28]. This can lead to overgrowth of *Candida *species and increased microbial translocation [7]. The difference in efficacy of SDD compared to SAP could possibly be explained by the fact that SDD reduces yeast load at the source of primary yeast colonisation (that is, the gastrointestinal tract).

The incidence of candidemia was significantly reduced by SAP, whereas the reduction in SDD studies was potentially relevant but not statistically significant. Furthermore, the meta-regression analysis revealed no significant difference between the pooled ORs of both types of prophylaxis, so no definite conclusions should be drawn. With a relatively high NNT of 38 for SAP and a relatively low incidence of candidemia of 3.8% in these critically ill patients, the prophylactic use of SAP in the general population of ICU patients does not seem justified, a reported high mortality rate of 25% to 60% [29] in patients with candidemia notwithstanding.

SDD led to a significant reduction in all-cause in-hospital mortality. The decrease in mortality rate may be due, at least in part, to the reduction of *Candida *infection by SDD, and antifungal prophylaxis indeed decreased mortality directly attributable to yeast. However, in the present review, SDD did not significantly reduce the rate of candidemia, as was shown in an earlier study [30]. A previous meta-analysis in critically ill patients showed that pneumonia and Gram-negative as well as overall bloodstream infections were reduced by SDD. [19,31], which would account for the decrease in mortality. The value of antifungal prophylaxis is likely to be much higher in individuals who receive antibacterial agents, and the reduced gastrointestinal microbial load may be a possible factor in the greater efficacy of SDD compared with SAP.

A systematic review of SDD in ICU patients found that mortality was reduced significantly in surgical patients only and not in medical patients. [19]. In the present review, adjustment for the proportion of surgical patients changed the difference in effect between the two types of antifungal prophylaxis for the outcomes of colonisation and candidemia. The difference in effect of SAP compared to SDD became smaller, but the direction of the difference was stable in favour of SDD. These results show that the effectiveness of prophylaxis is reduced with an increased proportion of included surgical patients.

The results of this review generally confirm the conclusions of earlier reviews examining the separate effects on yeast infections of SDD [30,32] or SAP [33-35]. However, these reviews have some shortcomings. The reviews on SDD/non-absorbable antifungal prophylaxis included pediatric studies [30,32] or liver transplant studies [30], which were excluded in the present review. Previous reviews on systemic prophylaxis included studies that included fluconazole studies only [33], studies on surgical patients only [34], or studies that examined groups with non-absorbable prophylaxis as control groups [35]. The present review is the first to examine the two methods of antifungal prophylaxis concurrently, showing the pros and cons of each regimen with respect to the outcomes of yeast colonisation, infection, candidemia, and hospital mortality.

The use of systemic (absorbable) drugs, like fluconazole, may have to be restricted for two reasons. First, antifungal drug use can initiate the possible emergence of azole-resistant strains. The present study did not investigate this aspect. Playford and colleagues [35] could find no significant increase of resistant strains in a review of absorbable antifungal prophylaxis. However, the pooled estimates on the emergence of resistant strains had wide CIs and could have suffered from insufficient power. Thus, no definite conclusions can be drawn from these observations. There is no compelling evidence to link prophylactic antifungal therapy with resistance, but it is quite clear that increased use of antifungal drugs has promoted the dissemination of azole-resistant fungi. This needs to be taken into consideration when considering the risk-benefit ratio for instituting widespread use of antifungal prophylaxis in critical care units. Second, systemic antifungal agents can have potential toxic effects. [35]. Systemic drugs may be advised as prophylaxis only in patients with increased risk of developing *Candida *bloodstream infections [18,23,28]. SDD may have fewer systemic side effects and thus can be given to critically ill patients to prevent *Candida *colonisation and infection. However, the effect of SDD use on resistance patterns of yeast is still a matter of debate. SDD may decrease the emergence of antibiotic resistance [36], but an increase in pathological bacteria (enterococci and coagulase-negative staphylococci) is of concern [37]. The matter of SDD-induced antifungal drug resistance is unclear.

Several factors have to be considered while interpreting the results of this review. First, most included studies had small sample sizes and the event rates of several outcomes were small. This means that individual studies had wide CIs, but even CIs around pooled ORs were still wide, and therefore the power for detecting clinically relevant differences for some outcomes was small. It means also that subgroup analyses have to be interpreted with care. Second, although the methodological quality of the studies was good on average, there is still room for improvement as only half of the studies applied blinding of treatment allocation and outcome assessment.

Third, studies used a wide variety of criteria for patient inclusion and exclusion. In addition, definitions of yeast colonisation and invasive yeast infection differed between studies as there is still no consensus on this subject. The value of the effect of prophylaxis on invasive yeast infection, especially, must be interpreted with scepticism because definitions vary between articles and it is debatable whether a mixed culture can be seen as evidence of an invasive infection. Unfortunately, not all articles published data on the culture results. The variation among definitions hampers the comparison of these outcomes between studies. In this review, we used widely accepted definitions and tried to redefine results if individual studies used other definitions. In particular, the definitions of prophylactic and pre-emptive treatment are often overlapping. Critically ill patients in the ICU are often already colonised or may be infected without being cultured, so prophylactic therapy can often be seen as pre-emptive. However, pre-emptive therapy will be given to a selected group of patients with a higher risk of yeast infections. There was one study that compared a pre-emptive strategy with a control group [38]. In the analysis of our data, we made no distinction between prophylactic and pre-emptive treatment strategies. Despite these differences in populations and definitions, the results for most outcomes were relatively homogeneous across studies with accompanying values of the I^2 ^statistic that were low.

Fourth, our review, like any other review, may have suffered from publication or selective reporting bias. Studies with a 'negative' result may be less likely get published, and results from non-significant outcomes are less likely to be reported [39,40]. The number of these file drawer studies exceeded the number of included studies in the analyses with positive results, reducing the unreliability of the validity of the outcomes. However, 14 studies provided antifungal therapy but were excluded for not scoring yeast/fungi. These studies did not state whether culture results were negative or whether no specific cultures were performed. This may cause pooled results of included trials, which are too optimistic.

## Conclusion

Both SAP and SDD antifungal prophylaxis strategies were effective in reducing yeast-associated disease across a range of critically ill patients. The reduction of yeast colonisation and infection was more pronounced in SDD studies compared with SAP, whereas candidemia was reduced foremost by SAP. SDD reduced all-cause in-hospital mortality, but both strategies reduced yeast-related mortality. Systemic drugs may be advised as prophylaxis in patients with a high risk of developing *Candida *bloodstream infections, whereas SDD may be given to critically ill patients to prevent *Candida *colonisation and infection.

## Key messages

• Antifungal prophylaxis reduces yeast-related morbidity and mortality.

• Selective decontamination of digestive tract (SDD) regimens are more effective than prophylactic regimens that include single antifungal drugs in reducing yeast colonisation, invasive yeast infection, and in-hospital mortality.

• The effectiveness of antifungal prophylaxis is inversely related to the proportion of included surgical patients.

• The incidence of candidemia is reduced by systemic antifungal prophylaxis but not by SDD.

## Abbreviations

ALI = acute lung injury; APACHE II = Acute Physiology and Chronic Health Evaluation II; ARDS = acute respiratory distress syndrome; CI = confidence interval; DF = degree of freedom; IQR = interquartile range; NNT = number needed to treat; OR = odds ratio; RCT = randomised controlled trial; SAP = single-drug antifungal prophylaxis; SDD = selective decontamination of the digestive tract; SICU = surgical intensive care unit.

## Competing interests

The authors declare that they have no competing interests.

## Authors' contributions

OvT participated in designing the study and in collecting and entering data. OvR and BL participated in designing the study. RW participated in collecting and entering data. JR participated in advising on statistical methodology. MB participated in designing the study and in advising on statistical methodology. All authors were responsible for critical analysis and interpretation of data. All authors read and approved the final manuscript.
